# Accelerometer-measured versus self-reported physical activity levels in women before and up to 48 months after Roux-en-Y Gastric Bypass

**DOI:** 10.1186/s12893-020-00699-7

**Published:** 2020-02-27

**Authors:** Sofie Possmark, Fanny Sellberg, Mikaela Willmer, Per Tynelius, Margareta Persson, Daniel Berglind

**Affiliations:** 1 Department of Global Public Health, Karolinska Institutet, K9, Social Medicine, 171 77 Stockholm, Sweden; 2 Department of Health and Caring Sciences, University of Gävle, 801 76 Gävle, Sweden; 3 Centre for Epidemiology and Community Medicine, Stockholm County Council, Box 45436, 104 31 Stockholm, Sweden; 4 Department of Nursing, Umeå University, 901 87 Umeå, Sweden

**Keywords:** Gastric bypass, Bariatric surgery, Physical activity, Accelerometer, Self-report

## Abstract

**Background:**

Roux-en-Y Gastric Bypass (RYGB) patients overestimate their time spent in moderate-to-vigorous physical activity (MVPA) to a greater extent post-surgery than pre-surgery. However, there is no data on discrepancy between self-reported and accelerometer-measured MVPA beyond nine months post-RYGB. The aim was to investigate how the duration of MVPA (main outcome) differs when comparing a self-administered questionnaire to accelerometer-data from pre-surgery and up to 48 months post-RYGB.

**Methods:**

Twenty-six (38%) RYGB-treated women with complete data from the original cohort (*N* = 69) were included. Participants were recruited from five Swedish hospitals. Mean pre-surgery BMI was 38.9 (standard deviation (SD) = 3.4) kg/m^2^ and mean age 39.9 (SD = 6.5) years. MVPA was subjectively measured by a self-administered questionnaire and objectively measured by the ActiGraph GT3X+ accelerometer at 3 months pre-RYGB and 9- and 48 months post-RYGB. Means and SD were calculated at 3 months pre- and 9- and 48 months post-RYGB. We calculated the *P*-values of the differences with Wilcoxon Signed-Rank test. For correlations between the self-administered questionnaire and the accelerometers, Spearman’s rank correlation was used.

**Results:**

Participants significantly overestimated (i.e. self-reported more time spent in MVPA compared to accelerometry) their MVPA in a higher degree post- compared to pre-RYGB surgery. Compared to pre-surgery, self-reported MVPA increased with 46.9 and 36.5% from pre- to 9- and 48 months, respectively, whereas changes were a 6.1% increase and 3.5% decrease with accelerometers. Correlations between self-reported and accelerometer-measured MVPA-assessments were poor at all measurement points (*r* = 0.21–0.42) and only significant at 48 months post-RYGB (*P* = 0.032).

**Conclusions:**

The discrepancy between self-reported and objectively assessed MVPA within the same individual is greater up to 48 months post-RYGB compared to before surgery. To help bariatric patients understand and hopefully increase their physical activity behaviors post-surgery, objective measures of physical activity should be used.

## Background

Engaging in physical activity provides several positive physiological and psychological health benefits [1]. Engaging in sufficient moderate-to-vigorous physical activity (MVPA) is especially associated with additional health outcomes and reduced mortality [2]. This is shown by the World Health Organization (WHO) as well as in new guidelines from the United States, that recommend a minimum of ≥150 min/week of MVPA for improved health outcomes [3, 4]. In addition, people who have lost weight and need to maintain a lower body weight are recommended to engage in > 300 min of MVPA per week, compared to the general recommendation of ≥150 min/week [3, 4]. Furthermore, there is some evidence suggesting that reduction of sedentary time (ST) can contribute to positive health outcomes [5, 6].

Bariatric surgery, such as Roux-en-Y Gastric Bypass (RYGB), is the most effective method for sustainable weight loss [7, 8]. Physical activity, and especially MVPA, after bariatric surgery is of importance as it helps improve surgical outcomes [9, 10], contribute to a greater weight loss [11], maintain the post-surgery weight loss, improve body composition [12–15], and increase cardiorespiratory fitness [13]. Moreover, there is some evidence of an association between sedentary time and fat-free mass loss post-surgery [16] and exercise post-surgery can attenuate the loss of fat-free mass and increase muscle strength [17, 18]. However, a recent meta-analysis did not find any associations between exercise and changes in lean body mass [15]. Previous studies, using self-reported questionnaires, have shown that bariatric surgery patients increase, often with over 20%, their time spent in MVPA post-surgery [9]. In contrast, when bariatric patients have worn objective physical activity measures, such as accelerometers, the results show that overall physical activity and MVPA do not increase [19–25], or increase to a small extent after surgery [26]. Only a few studies have evaluated the discrepancies between self-reported and accelerometer-measured physical activity pre- to post-surgery within the same individuals [19, 23, 27]. Bond et al. compared 25 bariatric patients’ self-reported physical activity to accelerometer measured physical activity pre- and six months post-bariatric surgery [23], and Berglind et al. compared self-reported and accelerometer measured physical activity three months pre- and 9 months post-RYGB in 43 women [19]. They both found that the patients significantly overestimated their self-reported MVPA at the post-surgery measurements, compared to pre-surgery, as the self-reported MVPA had increased post-surgery while the accelerometer-measured MVPA had hardly changed [19, 23]. All people overestimate their physical activity, but there is a larger over-reporting among individuals with overweight and obesity compared to normal population [28]. However, an interesting finding from the study by Berglind et al. [19] was that the same individuals overestimated their physical activity to a greater extent after their surgery compared to before: at 3 months pre-surgery the patients overestimated their MVPA with 7.5 min/day when comparing self-reported to accelerometer measured MVPA, while 9 months post-surgery the overestimation had increased to 26.2 min/day.

The aim of this study was to investigate how duration of MVPA (main outcome), and other intensities of physical activity, differ when comparing a self-administered questionnaire to accelerometer data pre- and up to 48 months post-RYGB, among 26 women undergoing RYGB-surgery.

## Material and methods

### Participants

Sixty-nine women between 28 and 52 years of age were recruited three months prior to undergoing RYGB surgery from five Swedish hospitals (Danderyd University Hospital, Ersta Hospital, S:t Görans Hospital, Uppsala University Hospital and Örebro Hospital). The women underwent surgery between June 2012 and January 2013. The researchers made baseline and subsequent follow-up home visits three months prior to RYGB, and nine and 48 months post-RYGB, respectively, where they weighed and measured the height of each participant. At the three measurement points, the women completed a self-administered questionnaire about their physical activity behaviors and wore the ActiGraph GT3X+ accelerometer for seven consecutive days. More detailed information about the recruitment and follow-up visits has been published elsewhere [19, 20]. A follow-up study, including 43 (62%) of the original 69 women who had complete questionnaire data and a valid accelerometer-measurement at the pre- and 9 months post-RYGB assessments, has been published previously [19]. The present study included 26 (38%) women who had complete subjective and accelerometer physical activity measures from pre-, 9- and 48 months post-RYGB measurement points.

### Self-administered questionnaire

At all measurement points, the participants completed a short self-administered questionnaire, intended to measure long-term total daily 24 h physical activity, about their average time and domain of physical activity during the previous week. The questionnaire has been validated against accelerometers in women [29] and against physical activity records (7-day physical activity diary) in men [30]. The questionnaire consists of six predefined activity time categories for “household work” (“less than 1” to “more than 8 h/day”), “walking/cycling’ (“hardly ever” to “more than 1.5 h/day”) and “work/occupation” (“mostly sitting down at work” to “heavy labor”), and five predefined activity time categories for “TV/reading” defined as leisure time inactivity (“less than 1 h/day” to “more than 6 h/day”) and “exercise” defined as leisure activity time (“less than 1 h/week” to “more than 5 h/week”). One question concerns occupational physical activity with six alternatives: “mostly sitting down”, “sitting down half the time”, “mostly standing up”, “mostly walking, lifts, carry little”, “mostly walking, lifts, carry a lot” and “heavy manual labor”. An open question is provided about number of sleeping hours per day.

### Accelerometer measurements

To objectively measure the time spent in physical activity at all measurement points, we used the ActiGraph GT3X+ accelerometer (ActiGraph, Pensacola, USA), which is a valid tool for estimating physical activity [31]. The participants were asked to wear the accelerometer during all waking hours on the right hip for seven consecutive days. Minimal wear time was defined as ≥10 h/day during ≥3 days [32]. We analyzed 3-dimensional vector magnitude (V_m_) activity counts, calculated as the square root of the sum of the counts of the three axes, which was recorded in 10-s epochs and aggregated to counts per minutes (cpm). Bouts and wear-time were computed using R packages “Accelerometry” and “Physical Activity”. Non-wear time was defined as 60 min with no counts, allowing for two minutes of non-zero interruptions [33]. Cut-offs to classify different intensities of physical activity were based on Santos-Lozano et al. [34]; sedentary time was set to any minute showing less than 100 cpm, light physical activity (LPA) as 101–3207 cpm and MVPA as more than 3208 cpm.

### Statistical analyses

From the predefined time categories in the self-administered questionnaire, we calculated the mean minutes per day and presented it as time spent in each activity domain. We calculated minutes per day spent in total MVPA by adding together active leisure time (walking/bicycling) and exercise. For the question about occupational physical activity, which did not have time categories, we grouped the alternatives “mostly sitting down” and “sitting down half the time” into a sedentary category, and the rest of the alternatives into an occupational physical activity category, to then calculate the prevalence. Means and standard deviations (SD) for the descriptive characteristics, accelerometers and variables from the self-administered questionnaire were calculated at 3 months pre- and 9- and 48 months post-RYGB. As the majority of all the variables was not normally distributed, we presented the mean differences between the three measurement points with non-parametric 95% bias-corrected and accelerated bootstrap (BCa) confidence intervals and calculated the *P*-values of the differences with Wilcoxon Signed-Rank test. To estimate if there was any correlation between the self-administered questionnaire and the accelerometers, Spearman’s rank correlation was used. Bland-Altman plots and scatterplots were conducted to visualize the results.

Finally, we performed sensitivity analysis comparing the included women (*n* = 26) to the women not included (*n* = 43) for baseline characteristics and the physical activity outcomes, as well as conducted sensitivity analyses among the women who wore the accelerometer for more than 12 h/day during more than 5 days and with complete data from the self-administered questionnaire at all three measurement points. All statistical analyses were conducted using Stata 14.1 (StataCorp) software.

## Results

### Descriptive characteristics

Descriptive characteristics of the 26 women with complete physical activity measures are presented in Table 1. The mean time intervals between the pre- to 9 and pre- to 48-months post-RYGB measurements were 12.0 (SD = 2.5) and 42.3 (SD = 4.6) months, respectively. At the time of surgery, mean age was 40.0 years (SD = 6.6). Four women (15.4%) had post-secondary or higher education and none had type 2 diabetes, which remained constant at all three measurement points. Change in body mass index from pre- to 9- and pre- to 48 months post-RYGB was − 11.8 (*P* < 0.001) kg/m2 and − 12.3 (*P* < 0.001) kg/m2, respectively. For the accelerometers, mean valid days of wear time (≥10 h/day) at pre-, 9- and 48-months post-surgery were 6.6 (SD = 0.9), 6.4 (SD = 1.0) and 6.8 (SD = 1.3) days, respectively, and mean hours of wear time were 14.5 (SD = 1.1), 14.8 (SD = 1.3) and 14.8 (SD = 1.3) hours/day, respectively. The majority had a sedentary occupation at the different measurement points, with prevalence of 61.5% (*N* = 16), 50% (*N* = 13) and 65.4% (*N* = 17) at pre-, 9- and 48 months post-surgery, respectively.

**Table 1:** Descriptive characteristics of the full cohort at baseline and of 26 women at 3 months pre- (T1) and 9- (T2) and 48 months (T3) post Roux-en-Y Gastric Bypass surgery (RYGB)

Characteristics	Pre-RYGB for the cohort (*N* = 69), Mean (SD)	T1: Pre-RYGB (*N* = 26), Mean (SD)	T2: 9 months post-RYGB (*N* = 26), Mean (SD)	T3: 48 months post-RYGB (*N* = 26), Mean (SD)
Age	38.8 (5.5)	39.9 (6.5)	40.9 (6.5)	43.4 (6.5)
Weight (kg)	107.4 (12.7)	106.8 (13.5)	74.1 (10.3)	72.2 (10.6)
BMI (kg/m^2^)	39.2 (3.3)	38.9 (3.4)	27.2 (3.0)	26.5 (3.2)
Waist circumference (cm)	117.9 (9.7)	116.5 (10.7)	87.0 (6.5)	87.8 (10.3)
Smoking (%)	22.1 (*N* = 15)	15.4 (*N* = 4)	7.7 (*N* = 2)	7.7 (*N* = 2)
Higher education (%)	11.6 (*N* = 8)	15.4 (*N* = 4)	15.4 (*N* = 4)	15.4 (*N* = 4)
Diabetes type 2 (%)	2.9 (*N* = 2)	0.0 (*N* = 0)	0.0 (*N* = 0)	0.0 (*N* = 0)

### Questionnaire versus accelerometers

**Fig. 1:**
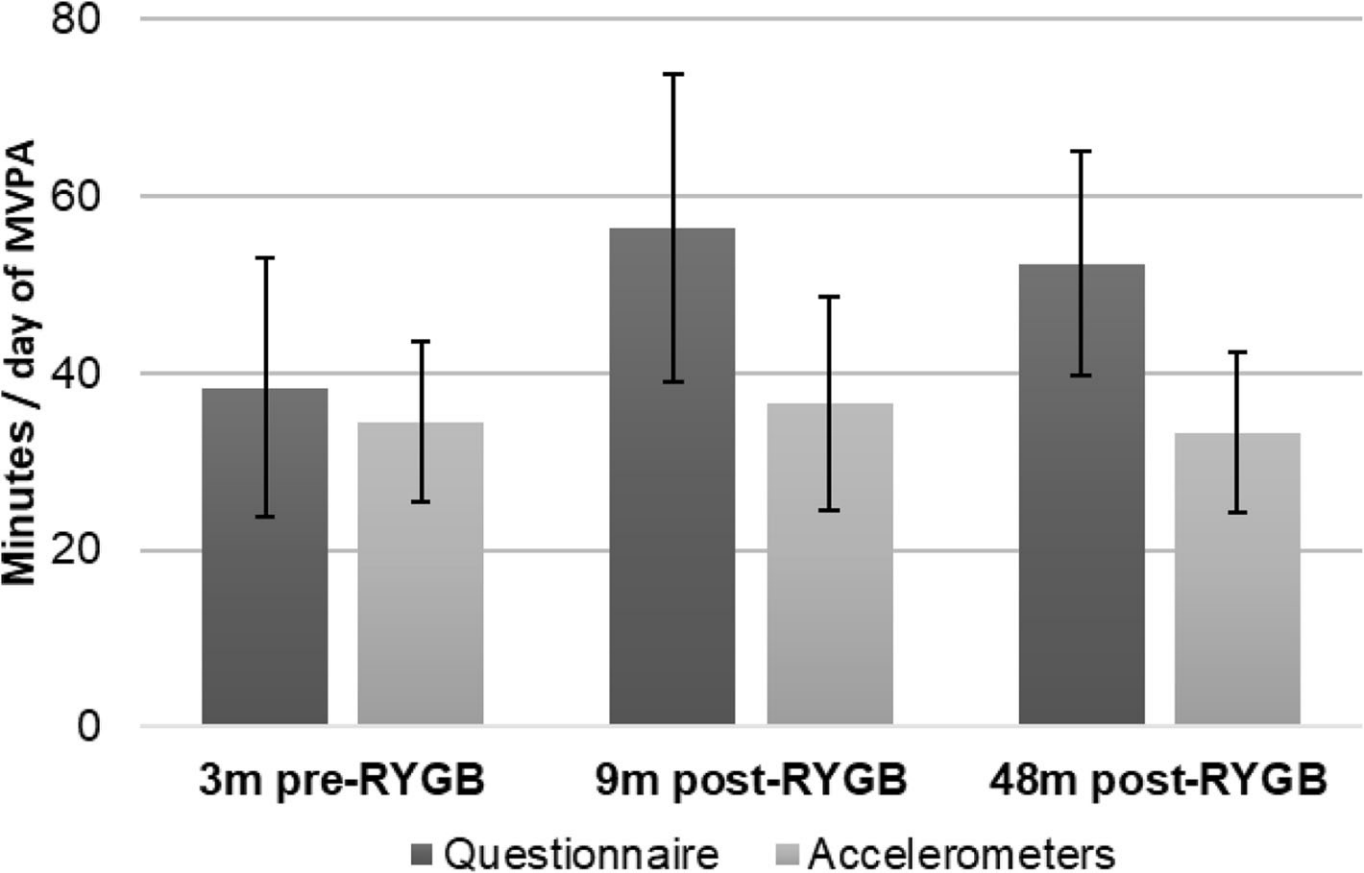
Self-reported versus accelerometer-measured moderate-to-vigorous physical activity (MVPA). Comparison between means of minutes per day of self-reported, measured by a self-administered questionnaire, versus objectively-assessed, measured by the GT3X+, MVPA in 26 women 3 months pre- and nine- and 48 months post Roux-en-Y Gastric Bypass surgery (RYGB)

Table 2 presents the means and differences of the various domains and intensities of physical activity for all the three measurement points. For the self-administered questionnaire, the domains “household work” and “total MVPA” (active leisure time plus exercise) increased statistically significant (all *P* < 0.005) from pre- to 9 months post-RYGB. The domain “exercise” had a significant increase at both 9- and 48 months post-RYGB, compared to pre-RYGB. The other domains did not differ statistically significantly from pre- to either of the two post-RYGB measurement points. Contrary, when physical activity was assessed objectively with accelerometers, there were no significant changes between any of the three measurement points, irrespective of physical activity intensity. For comparison, according to self-reported measurements, MVPA increased by 46.9 and 36.5% from pre- to 9- and pre- to 48 months post-RYGB, respectively. In contrast, the accelerometer-assessed MVPA only increased 6.1% from pre- to 9 months post-RYGB, while it decreased with 3.5% from pre- to 48 months post-RYGB. Fig. 1 illustrates the difference in means by the self-administered questionnaire and accelerometer-assessed MVPA.

**Table 2:** Physical activity by domain measured by a self-administered questionnaire, and intensities measured by the GT3X+ accelerometers, in 26 women 3 months pre- (T1) and 9- (T2) and 48 months (T3) post Roux-en-Y Gastric Bypass surgery (RYGB)

Variables (N = 26)	T1: Pre-RYGB Mean (SD)	T2: 9 months post-RYGB Mean (SD)	T3: 48 months post-RYGB Mean (SD)	Difference T1 to T2 (95% CI)^a^ *P*-value^b^	Difference T1 to T3 (95% CI)^a^ *P*-value^b^	Difference T2 to T3 (95% CI)^a^ *P*-value^b^
Questionnaire (minutes/day)						
Inactive leisure time (TV/reading)	131.5 (79.2)	129.2 (88.1)	120.0 (76.4)	−2.3 (−29.3–24.7) *P* = 0.963	− 11.5 (− 42.2–19.1) *P* = 0.366	−9.2 (− 43.3–24.8) *P* = 0.787
Household work	83.1 (54.5)	122.3 (76.3)	103.8 (72.6)	39.2 (16.6–61.9) *P* = 0.002	20.8 (− 6.6–48.1) *p* = 0.128	−18.6 (− 51.9–15.0) *P* = 0.121
Active leisure time (walking/bicycling)	29.6 (35.7)	39.4 (38.6)	34.6 (27.2)	9.8 (− 5.3–24.9) *p* = 0.109	5.0 (− 11.6–21.6) *P* = 0.254	−4.8 (− 20.1–10.5) *P* = 0.885
Exercise	8.7 (8.7)	17.0 (10.9)	17.8 (13.1)	8.2 (2.4–14.1) *P* = 0.004	9.1 (2.9–15.2) *p* = 0.002	0.8 (−5.6–7.2) *P* = 0.865
MVPA total (active leisure time + exercise)	38.4 (36.3)	56.4 (43.2)	52.4 (31.1)	18.0 (− 0.8–36.9) *P* = 0.047	14.1 (− 3.9–32.0) *P* = 0.091	−4.0 (− 21.7–13.7) *P* = 0.869
Accelerometers (minutes/day)						
MVPA	34.5 (22.6)	36.6 (30.2)	33.3 (22.6)	2.0 (−9.1–13.2) *P* = 0.869	−1.2 (− 11.2–8.7) *P* = 0.849	−3.3 (− 16.3–9.7) *P* = 0.929
Light physical activity	429.3 (96.9)	422.2 (80.3)	430.6 (101.6)	− 7.0 (− 39.1–25.0) *P* = 0.869	1.3 (− 28.6–31.2) *P* = 0.970	8.3 (− 23.4–40.1) *P* = 0.929
Sedentary time	406.4 (107.4)	431.4 (95.3)	424.2 (118.3)	24.9 (−4.8–54.6) *P* = 0.075	17.8 (− 15.5–51.2) *P* = 0.409	−7.1 (− 37.8–23.5) *P* = 0.354
Total physical activity	689.5 (228.3)	671.7 (243.3)	674.1 (244.6)	−17.8 (− 105.0–69.5) *P* = 0.304	−15.4 (− 101.3–70.5) *P* = 0.751	2.4 (− 96.9–101.7) *P* = 0.672

^*a*^*Confidence interval derived from paired T-test.*^*b*^*P-values between measurement points calculated with Wilcoxon Signed-Ranked test*

**Fig. 2:**
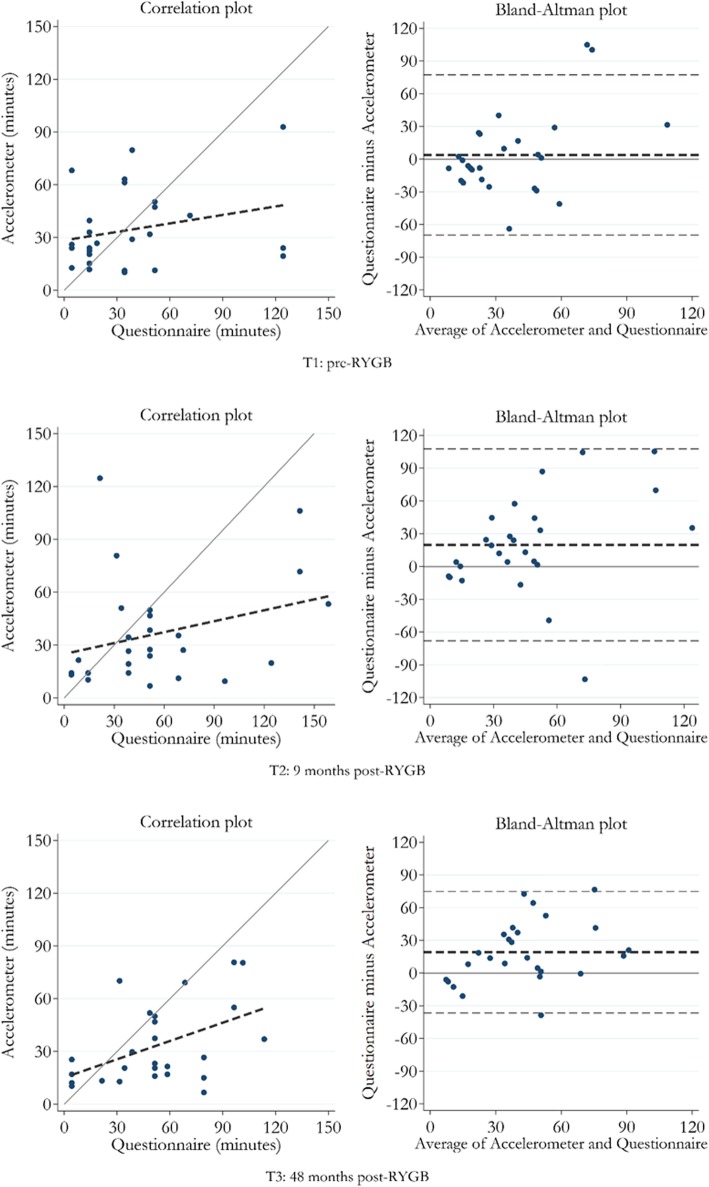
Bland-Altman plots and scatter plots of the correlation between the self-reported and objective measured moderate-to-vigorous physical activity (MVPA) at pre-RYGB (top), 9- (middle) and 48-months post-RYGB (bottom). Left side: Scatter plot with added 45-degree line (solid) indicating perfect agreement, and linear regression line (dashed). Right side: Bland-Altman plot with limits of agreement (±1.96*SD) and mean difference (dashed)

Table 3 presents the differences and correlations between the self-administered questionnaire and the accelerometer-assessed MVPA. The self-reported MVPA presented 3.8 (95% CI: − 11.3, 18.9) more min/day compared to the accelerometer-assessed MVPA at pre-surgery. At 9- and 48-months post-surgery, there was a statistically significant difference of 19.8 (*P* = 0.012) more min/day and 19.1 (*P* = 0.003) more min/day for the self-reported MVPA, compared to accelerometer-assessed MVPA, respectively. There was only significant correlation between the self-administered questionnaire and the accelerometer-assessed MVPA at 48 months post-RYGB (*r* = 0.42, *P* = 0.032), and correlations for all measurement points were poor (*r* = 0.21–0.42). The Bland-Altman plots shows that self-reported MVPA is consistently higher than the objectively measured MVPA over the whole range at both 9- and 48 months post-RYGB, and likewise shows no systemic differences at pre-RYGB (Fig. 2).

**Table 3:** Comparison between self-reported and objective measured moderate-to-vigorous physical activity (MVPA), measured by a self-administered questionnaire (domains: active leisure time + exercise) and accelerometers (GT3X+), in 26 women pre- (T1) and nine- (T2) and 48 months (T3) post Roux-en-Y Gastric Bypass surgery (RYGB)

Time points	Difference between questionnaire and GT3X+ (95% CI)^a^	*P*-value of the differences^b^	Correlation (*P-* value)^c^
T1: Pre-RYGB MVPA (minutes/day)	3.8 (−10.3–17.9)	0.970	0.21 (0.296)
T2: 9 months post-RYGB MVPA (minutes/day)	19.8 (3.3–36.3)	0.012	0.25 (0.213)
T3: 48 months post-RYGB MVPA (minutes/day)	19.1 (8.6–29.6)	0.003	0.42 (0.032)
	**Difference of differences (95% CI)**^**a**^	***P*-value of the differences**^**b**^	**Correlation (*P-*value)**^**c**^
Difference T1 and T2 MVPA (minutes/day)	16.0 (−3.3–35.3)	0.016	0.37 (0.062)
Difference T1 and T3 MVPA (minutes/day)	15.3 (−1.6–32.2)	0.082	0.32 (0.107)
Difference T2 and T3 MVPA (minutes/day)	−0.7 (− 17.5–16.1)	0.713	0.41 (0.035)

^*a*^*95% bootstrap (BCa) confidence intervals.*^*b*^*P-values calculated with Wilcoxon Signed-Ranked test.*^*c*^*Spearman’s rank correlation*

### Sensitivity analysis

We conducted sensitivity analysis for the descriptive and anthropometrical characteristics and found no significant differences between the women included from the original cohort (*N* = 69) and the women included in the present study (*N* = 26), see Table 1. At the original cohort, the prevalence of women with a higher education (11.6%, *N* = 8) was slightly lower compared to the 26 women included in the present study (15.4%, *N* = 4). There was a slightly higher prevalence of smokers in the original cohort (22.1%, *N* = 15), compared to the present study population (15.4%, *N* = 4), and two women (2.9%) in the original cohort had type 2 diabetes compared to none in the present study. We also conducted sensitivity analysis comparing the women included in the study versus the women not included from the original cohort (*n* = 43), but found no significant differences on any of the baseline characteristics or the physical activity outcomes (*P* > 0.05). We also conducted all analyses presented in Tables 2 and 3 with women (*N* = 21) who wore the accelerometer for more than 12 h/day during more than five days and who had complete data from the self-administered questionnaire at all three measurement points. There were no significant differences in the results that changed any of our conclusions (data not shown).

## Discussion

### Main findings

The purpose of the present study was to investigate how intensities of physical activity differ when comparing self-administered questionnaires to accelerometer measurements pre- and up to 48 months post-RYGB, among 26 women treated with RYGB. To our knowledge, our study is the first to have investigated physical activity measurement discrepancies up to 48 months post-RYGB within the same individuals. Our results show that the discrepancy between self-reported and objectively measured MVPA was greater up to 48 months after, compared to before RYGB. Self-reported MVPA was substantially greater after surgery, while objectively measured MVPA remained unchanged. Correlations between the self-administered questionnaire and the accelerometer-measured assessments of time spent in MVPA was poor (*r* = 0.21–0.42) at all measurement points and only significant at 48 months post-RYGB (*P* = 0.032).

### Previous research

The present study is one of only a few that have measured physical activity subjectively and objectively at both pre- and post-bariatric surgery in the same individuals [19, 23, 27]. The previous study by Berglind et al., that included 43 women from the cohort used in the present study, showed that the participants overestimated their time spent in MVPA both before and 9 months after RYGB, but the overestimation was larger post-RYGB compared to pre-surgery [19]. The study by Bond et al. compared self-reported to objectively measured MVPA at pre- and six months post-surgery in 25 patients undergoing bariatric surgery and found the same conclusion as Berglind et al.; that there was a greater overestimation of the self-reported physical activity post-surgery than pre-surgery [23]. Our results confirm that the overestimation of MVPA post-bariatric surgery, compared to pre-surgery, persists and remains greater up to 48 months post-surgery. In contrast with this, a study by Afshar et al. with bariatric surgery patients showed no change in any physical activity intensities from pre- to six months post-surgery, neither in subjective nor objective physical activity measurements. However, 45% of the study participants had pre- and post-surgery reported long-term illnesses, physical or mental health problems or disabilities, which might have affected their ability to be physically active [27]. A study by Bergh et al. showed a high prevalence of overestimating the time spent in MVPA, assessed by self-administered questionnaire, compared with objective-measured MVPA up to 24 months post-surgery, but, no objective data at pre-surgery were available to be able to compare the difference from pre- to post-surgery. However, their results showed a high over-reporting of time spent in MVPA, where 80% of the participants reported to have met the weekly MVPA guidelines, while according to objective measures the prevalence was only 17.9% [24]. People with obesity tend to overestimate their MVPA levels [28, 35] and also misclassify the intensity of physical activity to a higher extent than people of normal weight [35]. This might to a certain degree explain why bariatric patients overestimate their time spent in MVPA, shown in this and other studies described above, but why the overestimation increases to such a large extent post-surgery compared to pre-surgery is unknown. One hypothesis is that patients feel that everything in their daily life becomes easier after surgery, as they lose weight and gain more energy and mobility [36–39]. As everything becomes easier, their subjective feeling is that they are more physically active, when in reality, their physical activity behavior remains unchanged. Several interview studies with bariatric patients report that after surgery, patients experience increased physical function and motivation for being physically active [36, 37]. As obesity-related pain decreases [39], they can walk without being afraid of falling and also mentally feel much more satisfied and relieved at being able to be more physically mobile [38]. This could contribute to bariatric patients’ beliefs that they have increased their MVPA. A study by Guo et al. found that objectively-measured physical activity had a twofold stronger relationship to adiposity and other health outcomes, compared to self-reported physical activity [40]. This strengthens the importance of measuring physical activity, and especially MVPA, in an objective manner as part of the post-surgery support. Thus, for both post-surgery care as well as for future research, questionnaires asking about specific activities could be of important value, if used together with objective measurements, as a way to get a deeper understanding and knowledge of what type of activity has been performed, which could make an addition when interpreting the data.

### Strengths and limitations

The present study has several strengths. This study has a longitudinal design and it is, to our knowledge, the only study of this kind that has reported the difference between subjectively and objectively measured MVPA on RYGB patients from pre-surgery up to 48 months post-surgery, within the same individuals. With the longitudinal design and when comparing subjective to objective data we can control for factors that are constant during the measurement points (e.g. age and gender). We used the ActiGraph GT3X+ accelerometers to objectively measure participants MVPA, which is a tool that accurately estimates physical activity in free-living subjects [31]. Three to four days of wear time is sufficient for achieving 80% reliability of MVPA [41], and we chose to use an inclusion criterion for wear time of at least three days with a minimum of 10-h wear time per day. To include at least one weekend day was not a requirement, as our aim was to compare in-between data of self-reported to objectively-measured MVPA, and not to estimate the physical activity levels per se [42]. The self-administered questionnaire does not accurately capture levels of physical activity. Thus, an advantage is that it can capture different types of activities that an accelerometer is unable to measure, such as household activities or occupational physical activity [29].

This study has some limitations. Our sample is quite homogenous with respect to, for example, age, gender and education level, which may affect the possibilities to generalize the results to other population samples (e.g. males) and our sample is quite small. Accordingly, it might provide results of limited significance or limit the power of our results. Also, only patients who underwent RYGB were included, which should be considered if results are compared to other types of bariatric surgery. Due to the exclusion of participants who lacked complete data from the self-administered questionnaire and valid accelerometer measures for all three measurement points, only 38% of the original cohort was included. This might have resulted in selection bias and have affected the results. However, we conducted sensitivity analysis between the original cohort (*N* = 69) and the present study participants (*N* = 26) for the descriptive and anthropometrical characteristics, and there were no significant differences. The self-administered questionnaire does not specify what kind of physical activity is performed under the predefined activity time category “Exercise”, as it only specifies duration in an activity and not the intensity of the exercise. This could result in inaccurate comparisons to the accelerometer if a participant, for example, is practicing yoga as her “exercise” but it is not registered in the accelerometer as “MVPA”. The ActiGraph GT3X+ may not be able to differentiate between standing or sitting, which might produce inaccurate estimates of sedentary time [43]. There are also activities where the GT3X+ cannot be used, for example swimming. Also, doubly labeled water have been shown to more accurately estimate physical activity energy expenditure, compared to accelerometers [44], but we measured and compared the *time* spent in different activities and not the energy expenditure. For this reason, accelerometers were chosen as the most appropriate tool. Finally, the GT3X+ has not been validated in RYGB patients.

## Conclusion

This study shows that the discrepancy between self-reported and objectively assessed physical activity is greater up to 48 months compared to before RYGB within the same individual. This novel finding adds further evidence that women overestimate their levels of physical activity to a greater extent after, compared to before RYGB, and that the overestimation persists long-term. Our findings highlight the importance of using objective measures when investigating physical activity among bariatric patients both pre- and post-surgery, in order to fairly estimate physical activity behaviors. As previously mentioned, sufficient levels of MVPA are important post-surgery to improve the surgical outcomes as well as maintaining the weight loss [9, 10, 12, 16]. Therefore, it is of importance to give bariatric patients a tool for estimating and understanding their own physical activity behavior, as bariatric patients might not increase their physical activity behavior or motivation for physical activity if they already “think” that they are sufficiently active. These tools may include accelerometers provided by the hospitals as a part of the after-surgery care, that health-care personnel can analyze and discuss with the patient at a follow-up visit. Hospitals may also organize exercise groups for bariatric surgery patients post-surgery, where patients can get familiarized with different types of physical activity in a safe environment together with other patients that share the same experience (having undergone a bariatric surgery). Patients may also be encouraged to use mobile apps or own advices that measures physical activity (like pedometers, smart watches or apps that continuously measures daily physical activity) to learn their own physical activity behavior and to see how different activities are registered in different physical activity levels. They may also get information about the existing physical activity guidelines and what advantages sufficient physical activity can have on their health as well as how physical activity can optimize their results of the surgery [15, 45]. As concluded, bariatric patients highly overestimate their physical activity up to 48 months after surgery, and therefore accelerometers may play a role in helping patients to understand and, hopefully, increase their physical activity behaviors post-surgery.

## Data Availability

The datasets used during the current study are available from the corresponding author on reasonable request.

## Abbreviations

BMI: Body mass index
LPA: Light physical activity
MVPA: Moderate-to-vigorous physical activity
RYGB: Roux-en-Y gastric bypass
ST: Sedentary time
